# Possible interference of *Bacillus thuringiensis* in the survival and behavior of Africanized honey bees (*Apis mellifera*)

**DOI:** 10.1038/s41598-021-82874-1

**Published:** 2021-02-10

**Authors:** Gabriela Libardoni, Pedro Manuel Oliveira Janeiro Neves, Raiza Abati, Amanda Roberta Sampaio, Fabiana Martins Costa-Maia, Edgar de Souza Vismara, Everton Ricardi Lozano, Michele Potrich

**Affiliations:** 1 Laboratório de Controle Biológico (LABCON), Universidade Tecnológica Federal Do Paraná (UTFPR), Campus Dois Vizinhos (DV), Estrada para Boa Esperança, Km 04, Dois Vizinhos, Paraná, CEP 85.660-000 Brazil; 2 Programa de Pós-Graduação em Agronomia, Universidade Estadual de Londrina (UEL), Londrina, Paraná Brazil; 3 Programa de Pós-Graduação em Agroecossistemas (PPGSIS), UTFPR-DV, Dois Vizinhos, Paraná, CEP 85.660-000 Brazil; 4 Programa de Pós-Graduação em Zootecnia (PPGZO), UTFPR-DV, Dois Vizinhos, Paraná, CEP 85.660-000 Brazil

**Keywords:** Entomology, Environmental impact, Ecosystem services

## Abstract

*Bacillus thuringiensis* (Bt), an entomopathogenic bacterium, has been used as bioinsecticides for insect pest control worldwide. Consequently, the objective of this work was to evaluate the possible effects of commercial formulations of Bt products, Dipel and Xentari, on the survival and behavior of Africanized honey bees (*Apis mellifera*). Bioassays were performed on foragers and newly emerged (24-h-old) bees that received the products mixed in the food. Their survival and behavior were evaluated through the vertical displacement tests and the walk test, analyzed using software Bee-Move. Then, histological analysis of the mesenterium was performed. As control treatment was used sterile water. The honey bees’ survival was evaluated for between 1 and 144 h. No interference of *B. thuringiensis*, Dipel and Xentari, in the survival of Africanized honey bees were found. Only Xentari interfered with vertical displacement behavior of newly emerged (24-h-old) bees. Both the products tested were selective and safe for *A. mellifera*.

## Introduction

The species *Apis mellifera* L. (Hymenoptera: Apidae) has a wide distribution and generalist foraging. The honey bees can be a biological indicator of environmental pollution and, the use of them as a monitor also contributes to the ecological impact statement on the presence of agrochemicals, especially pesticides^1,2^. It is a pollinating species of great importance, as it can visit twice as many flowers as other bees^3^. In addition, they are responsible for the increased productivity and higher quality of fruits from various agricultural crops, such as apple, cherry, tomato, melon, coffee, cocoa, and soybean^4–6^. The economic contribution of pollinators, in Brazil, is 30% (US$ 12 billion) of the total annual agricultural income of the dependent crops (totalizing almost US$45 billion)^4^. The honey bee *A. mellifera* has a direct and indirect contribution in this scenario, as pollinating agent worldwide^4,7^.

However, since 2006, there have been reports of rapid weakening or loss of colonies, giving rise to a phenomenon now defined as colony collapse disorder (CCD)^8^. One of the main factors associated with CCD is the contamination of bees by synthetic phytosanitary products that are used widely in agricultural crops, mainely for the control of insect pests^9,10^. In Brazil, another emerging problem is the mortality in whole hives or parts of hives. Often, hundreds of dead bees with signs of intoxication are observed near the hives, owing to the use of pesticides^11^.

During foraging, the workers may come into contact with several plants with contaminated or treated flowers, and the active ingredients of the insecticides, even if they do not cause immediate death of bees, can negatively affect their orientation and flight capacity. This may make it difficult for the bees to return to the colony, or, even they manage to return, they bring these active ingredients back to the colony, which may weaken the colony or even cause it to die^12–15^.

Biological control, an alternative to chemical control, uses organisms, commonly called “natural enemies”, which keep the population density of an insect pest below the level that can cause economic damage^16^. This control method is considered to be safer for non-target organisms, such as pollinators^17^. Among the different biological control agents, the bacterium *Bacillus thuringiensis* (Bt), is considered successful^18^ and efficient and is recommended for the control of insects, including those of Lepidoptera, Coleoptera, and Hymenoptera^19–23^. In general, the side effects of a biological agent vary for different organisms, owing to the greater specificity for the target organism. This characteristic is advantageous, making it the safest solution for the management of insect pest populations. However, as it is important to understand all the possible effects of these agents, indirect and constant monitoring may be needed to ensure that they do not cause damage to non-target insects, such as bees^24^.

Bt-based products are used for pest control in various crops visited by *A. mellifera* and this may lead to exposure of workers to the Bt products during foraging. Contact can occur during spraying in the field or through the ingestion of contaminated nectar and pollen. In addition, honey bees can also ingest the bacteria while cleaning themselves. As the mode of action of the bacteria is by ingestion and there is the possibility that these agents will be ingested by honey bees, the objective of this work was to evaluate the possible interference of the commercial formulated products containing *B. thuringiensis* on the survival and behavior of Africanized honey bees (*A. mellifera*).

## Results

### Survival bioassay

**Figure 1:**
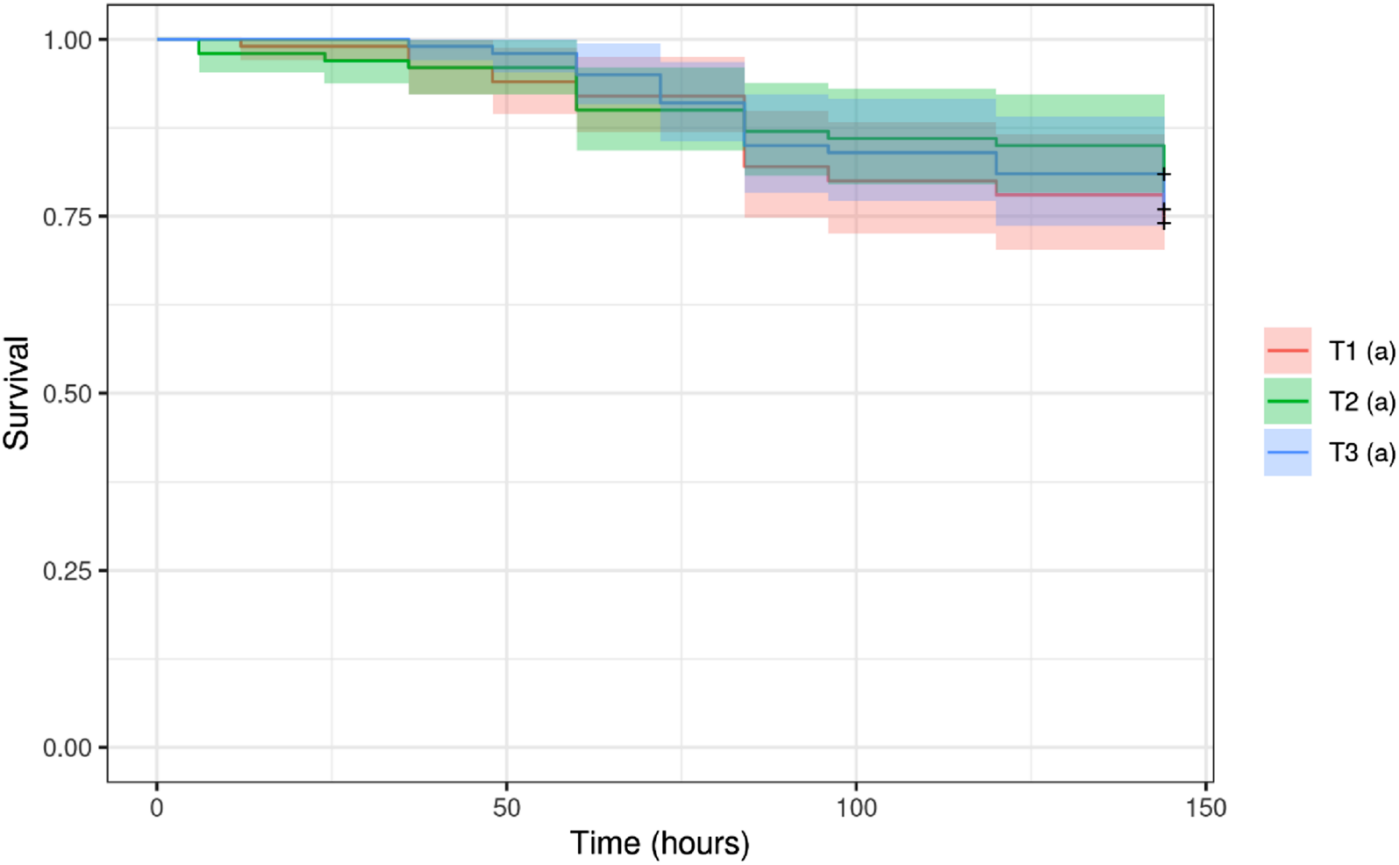
Graph of the survival of newly emerged (24-h-old) worker bee of Africanized *Apis mellifera*, by Kaplan–Meier, adjusted to the period (h) after feeding with Candi paste incorporating commercial products (T2, Dipel; T3, Xentari) or the control diet (T1). The bees were kept in controlled environment (26 °C ± 2 °C, RH, 60% ± 10%; 12-h photoperiod). The same letters indicate that there was no significant difference between the results (*p* < 0.05).

There was no difference in survival of newly emerged (24-h-old) honey bees when fed with Candi paste containing Dipel or Xentari. After 144 h, the survival rates of bees were 74%, 80%, and 76% for the control treatment (Candi Paste without treatment), treatment with Dipel, and treatment with Xentari, respectively (Fig. 1).

**Figure 2:**
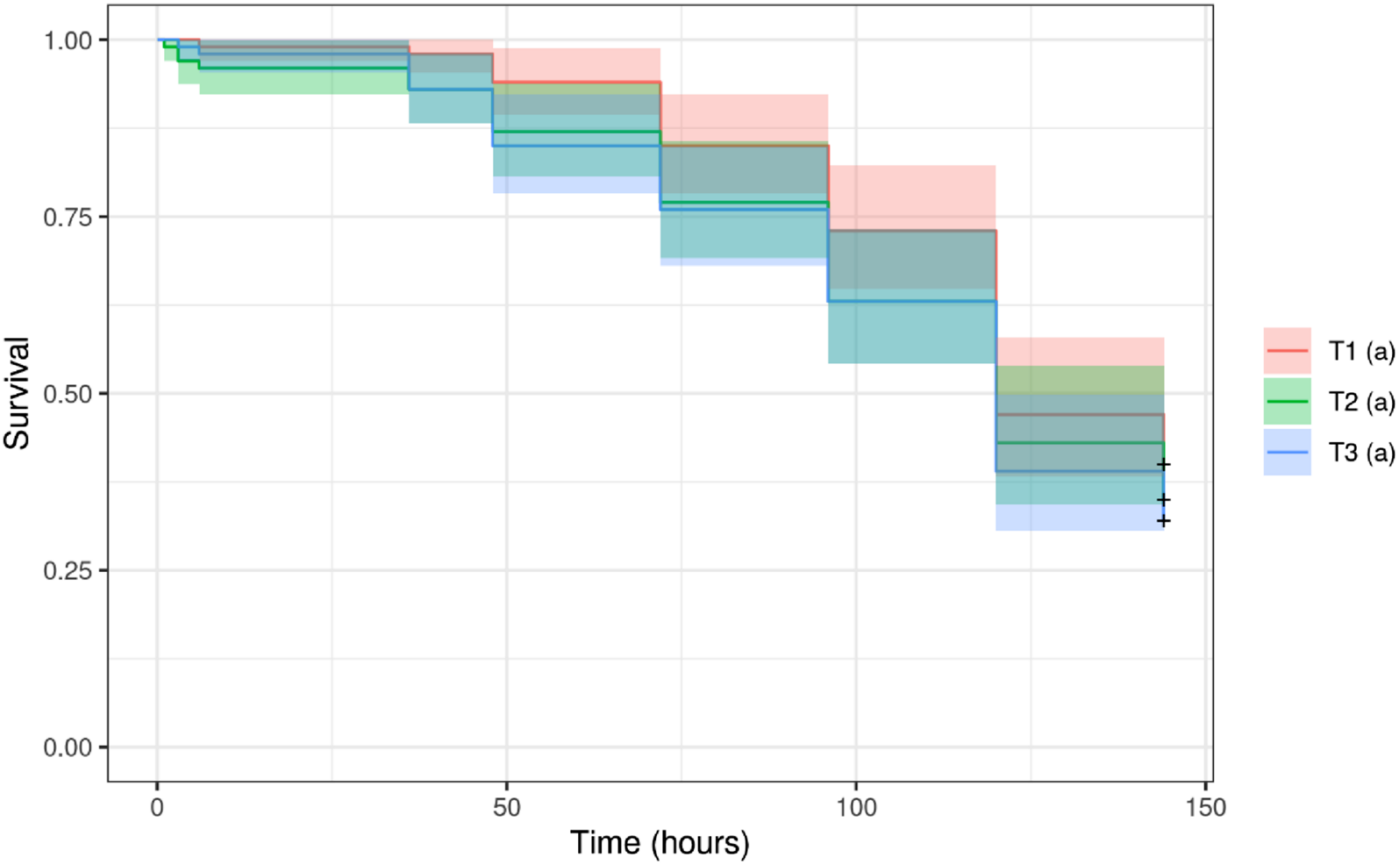
Graph of survival of foragers bees (*A. mellifera*)*,* by Kaplan–Meier, adjusted to the period (h) after feeding with Candi paste incorporating commercial products (T2, Dipel; T3, Xentari) or the control diet (T1). Temperature (26 °C ± 2 °C, RH 60% ± 10%; 12-h photoperiod). The same letters indicate that there was no significant difference between the results (*p* < 0.05).

In survival bioassay of foragers bees, differences were also not observed for bees fed with Candi Paste containing either Dipel or Xentari or the control (Fig. 2).

### Vertical displacement bioassay

**Figure 3:**
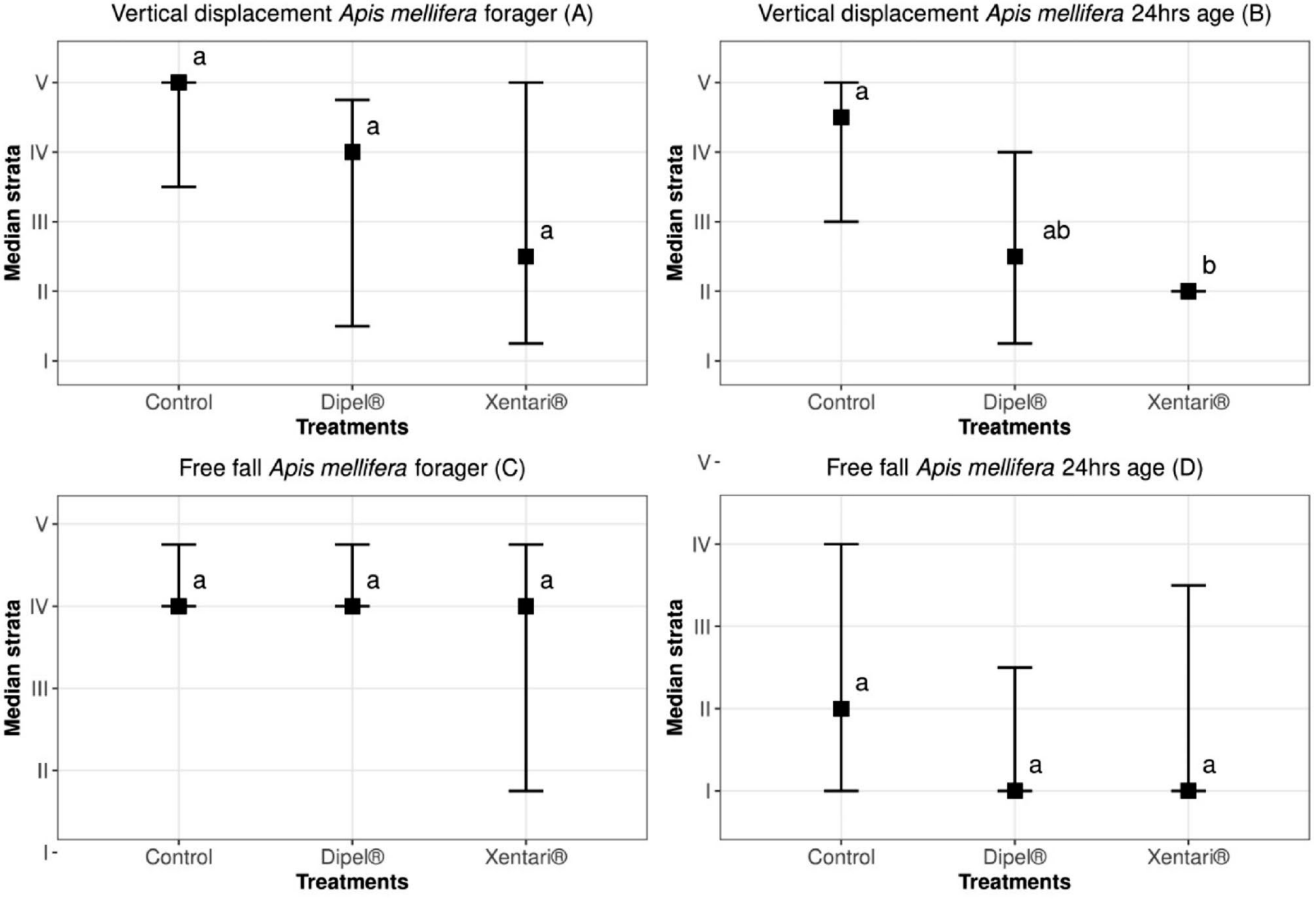
Vertical displacement (**A**,**B**) and free fall (**C**,**D**) of foragers and newly emerged (24-h-old) worker bees (*Apis mellifera*), 144 h after ingestion of Candi paste incorporating the indicated treatments. The squares represent the median strata values for each treatment with the respective first and third quartiles. The same lowercase letters within the figures indicate that there was no significant difference between treatments (*p* < 0.05) for the multiples comparison Tukey test.

Only the newly emerged (24-h-old) worker bees fed with Candi paste containing Xentari had reduced vertical displacement and were unable to reach the highest levels in the tower (Fig. 3B). The foragers bees in vertical displacement bioassay (Fig. 3A) and the foragers and newly emerged (24-h-old) worker bees of free fall bioassay (Fig. 3C,D) did not present statistical differences between treatments at a significance level of 5%.

It was not possible to compare the vertical displacement between foragers bees and newly emerged worker bees, nor the free fall bioassay. This is because the bioassay with foragers bees and newly emerged worker bees were carried out on different days, since they come from the same hives (same frames), standardized and marked for bioassays.

This bioassay was carried out to simulate field situations, where bees ingest food containing Bt and need to fly back to the colonies, or in search of new food sources, being possible to verify changes in flight behavior when in contact with Bt.

### Walking bioassay

**Figure 4:**
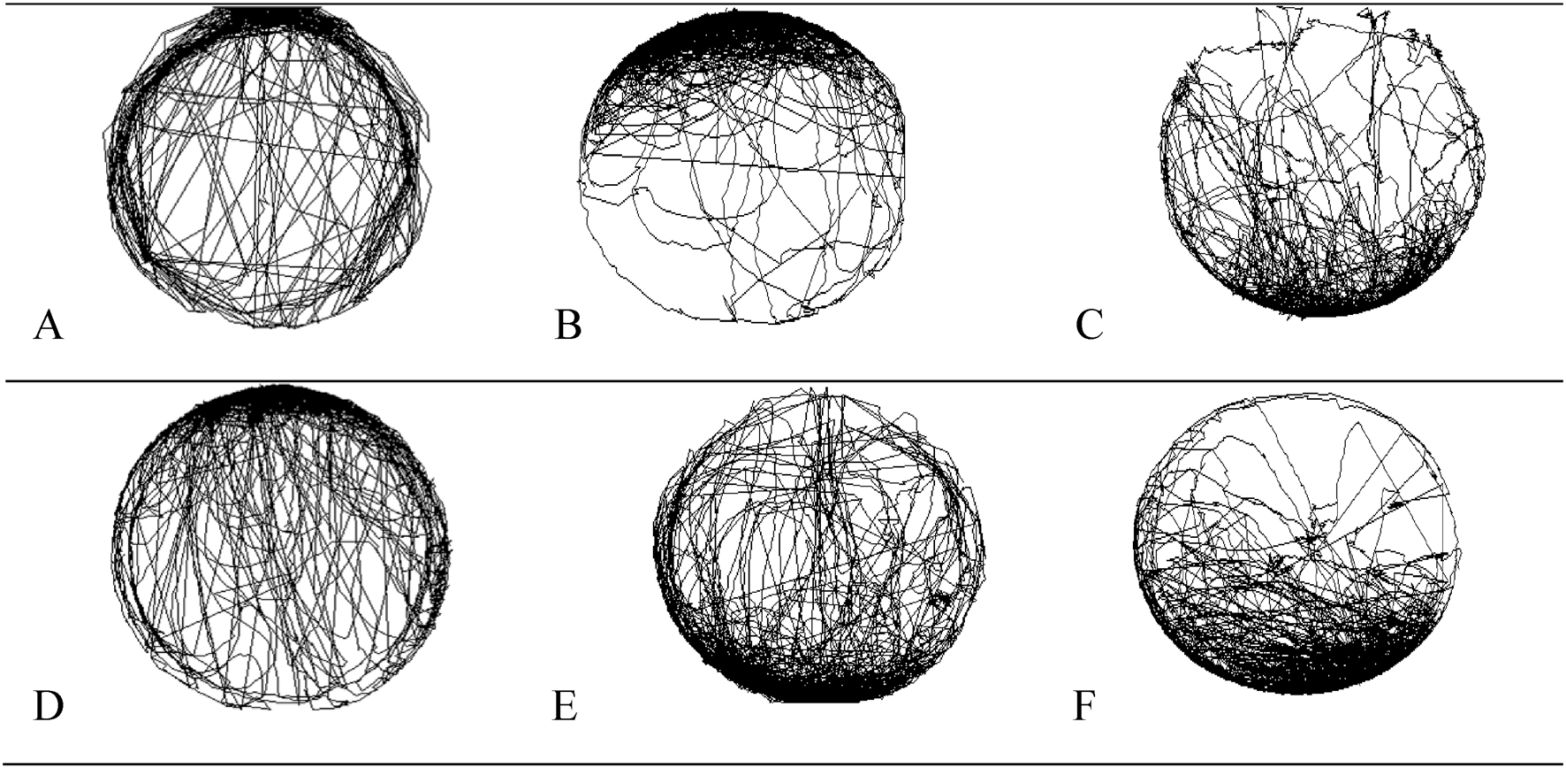
Path taken by *Apis mellifera* bees at 24 h after ingestion of Candi paste incorporated with the indicated products. (**A**) *A. mellifera* newly emerged worker bees (control); (**B**) *A. mellifera* newly emerged worker bees (Dipel); (**C**) *A. mellifera* newly emerged worker bees (Xentari); (**D**) *A. mellifera* foragers worker (control); (**E**) *A. mellifera* foragers worker (Dipel); (**F**) *A. mellifera* foragers worker (Xentari).

The average speed of the honey bees, the distance covered, the walking time, and the resting time were not affected by Candi paste incorporated with the products (Table 1 and Fig. 4). This bioassay was carried out to evaluate possible effects of the products ingested by the bees on the movement capacity.

**Table 1:** Data showing the average speed of the honey bees (mm/s), distance covered (mm), walking time (s), and the resting time (s) of foragers and newly emerged (24-h-old) worker bees at 24 h after ingestion of Candi paste incorporated with the products [± standard error (SE)].

	Treatment	Average speed (mm/s) ± SE	Distance covered (mm)	Resting time (s)	Walking time (s)
Newly emerged (24-h-old) workers	Control	32.5 ± 4.7a	16,931.0 ± 3527.1a	182.5 ± 61.1a	417.5 ± 61.1a
	Dipel	32.3 ± 3.4a	10,994.0 ± 2381.3a	189.5 ± 48.1a	410.5 ± 48.1a
	Xentari	35.2 ± 3.5a	13,007.4 ± 2617.3a	184.2 ± 48.1a	427.4 ± 43.5a
Foragers workers	Control	21.7 ± 3.6A	12,469.1 ± 1842.0A	146.3 ± 19.9A	453.8 ± 19.9A
	Dipel	20.5 ± 2.6A	12,562.0 ± 1474.1A	124.8 ± 11.2A	475.2 ± 11.2A
	Xentari	34.5 ± 5.7A	13,771.8 ± 3294.8A	151.0 ± 24.9A	449.0 ± 24.9A

The same lowercase letters in the column indicate that there was no significant difference (*p* < 0.05) for the foragers bees. The same uppercase letters in the column indicate that there was no significant difference (*p* < 0.05) for the newly emerged (24-h-old) worker bees.

### Histological analysis

The mean villi length of the newly emerged and forager workers fed with the commercial products Dipel and Xentari was between 109.4 μm and 123.9 μm, which was not significantly different from the control treatment (Table 2).

**Table 2:** The mean length of villi of the mesenterium [± standard error (SE)] of *Apis mellifera* bees after 24 h of ingestion incorporated with products.

Treatment	Average length of Villi ± SE (μm)	
	24-h-old worker bees	Foragers bees
Control	113.9 ± 3.3a	117.1 ± 5.2a
Dipel	109.4 ± 4.2a	123.9 ± 7.1a
Xentari	110.3 ± 1.8a	122.0 ± 11.3a

The same lowercase letters in the column indicate that there was no significant difference (*p* < 0.05).

## Discussion

The pathogenicity of *B. thuringiensis* depends on the ingestion of bacterial proteins by the insect. The Cry proteins function as endotoxins that, upon contact with the midgut of insects, are solubilized, activated and act in the intestinal villi, causing an ionic imbalance, that resulting in the formation of pores and consequent destruction of the digestive tract and death of the insect^25,26^.

In this study, we analyzed that Dipel and Xentari did not negatively affect newly emerged (24-h old) worker and forager Africanized honey bees (*A. mellifera*). Control bees showed mortality because bees are social insects and need the pheromones released by the queen bee to control activities. As there were no queen bees in the bioassays and only workers, this causes a considerable mortality rate. In addition, forager bees are older than 24 h-old workers (young worker/emerged worker), which is why their mortality rate is higher^27^. There are several strains of *B. thuringiensis* that are capable of producing different proteins; which toxicity varies according to the order and species of insect. Each strain produces one or more specific toxins that determine its toxicity; thus, the specificity of the host of each strain^28,29^ can make the same bacteria selective for some insects and non-selective for others. Results of the selectivity of the Cry1Ba protein has been shown, as there was no difference in the estimated survival for Africanized bees (*A. mellifera*) that received the protein in their food source and those that did not (control)^30^. Italian bees *A. mellifera* fed with pollen containing proteins Cry1Ba3 did not show significant differences in survival, pollen consumption, weight, detox enzyme activity between treatments^31^.

The survival of *A. mellifera* worker bees fed with a product based on *B. thuringiensis* var. *aizawai* and *kurstaki* at different concentrations (field dose 100.0 g/hL; low concentration, 40.00 g/hL; and very high concentration, 24,400.00 g/hL); selectivity was found at low concentration (40.00 g/hL) because it did not induce the death in Africanized honey bees at the end of 96 h^32^. The field dose (100.0 g/hL) caused resulted in a mortality rate of 5%, which was not significantly different from the control, whereas the high concentration (24,400.0 g/hL) resulted in a mortality rate of 15% at the end of the experimental period, which was significantly different from the control^32^. In other studies, the comparison of three strains of *B. thuringiensis* (IPS 82, BR 81, and BR 147), all reduced the survival of Africanized honey bees (*A. mellifera*) workers when incorporated into the diet^33^ owing to the mode of action of bacteria in insects.

In our study, Xentari treatment decreased the flight capacity of the 24-h-old worker bees in the vertical displacement assay; they achieved lower flight heights and had difficulties reaching the light source at the top of the tower. It has been emphasized that younger honey bees, when they are exposed to products, may be more negatively impacted; for example, more severe effects on the flight behavior of honey bees have already been observed for methyl benzoate^34^, imidacloprid^35^, pyriproxyfen, chlorantraniliprode, and azadirachtin^36^. Changes in flight behavior influence the collection of pollen and nectar, affecting the entire development of the colony, as well as the pollination of the surrounding crops.

Despite these results, it was found that the products Xentari and Dipel did not interfere in survival of Africanized honey bees *A. mellifera* (24-h old and foragers). *Bacillus thuringiensis*, in other tests, was also shown to be safe for *A. mellifera*, not causing mortality^37^ and, did not change the visitation behavior of these pollinators to soybean flowers^38^ or locomotor activity^39^, corroborating the results of our study, which also did find no change behavior of insects that were exposed to the bacteria. Besides that, it was observed that some isolates from *B. thuringiensis* do not negatively affect adult and larvae of worker bees from *A. mellifera*^37^, not causing sublethal effects such as the larvae development, the food consumption, and proboscis’s extension response in adults^39^.

Dipel is formulated with *B. thuringiensis* subsp. *kurstaki*, containing the proteins Cry1Aa, Cry1Ab, Cry1Ac, Cry2Aa, and Cry1Ac, whereas Xentari is formulated from *B. thuringiensis* var. *aizawai*, which produces Cry1Aa, Cry1B, Cry1Ca, and Cry1Da^40^ which may be one factor related to the differences observed in the flight capacity of 24-h-old worker bees. When Cry1Ba was present in *A. mellifera* feed, no changes were observed in survival time^30,41^, consumption of the food^41^, flight^30^ or time of flight^30^.

In the present work, it was not possible to verify differences between the villi length in the mesenterium of honey bees fed with the different products/concentrations. A similar result was observed when Africanized adult honey bees (*A. mellifera*) were fed diets containing *B. thuringiensis* var. kurstaki HD-1^42^ and in studies with larvae that were fed diets containing *B. thuringiensis* proteins (Cry1C or Cry2A)^43^. However, D’Urso et al.^32^ found that changes in the intestine had occurred at 96 h after treatment with *B. thuringiensis*. Some acute effects may occur in the long term in the intestinal epithelium of the bees that have ingested Bt, despite the apparent absence of toxicity (i.e., no alteration in survival of the bees). This may mask other physiological disruptions that are harmful to bees, particularly in the case of exposure to biological products in combination with other environmental stressors^32,44^.

Intestinal bacteria from nursing bees (*A. mellifera carnica*) fed with Bt corn pollen, that expresses three insecticidal Cry proteins (Cry1A.105, Cry2Ab2 and Cry3Bb1), did not showed difference^45^, just as the Cry1Ie toxin did not modify the midgut bacteria of worker bees *A. mellifera ligustica* and *Apis cerana cerana* under laboratory conditions^46,47^. In this same way, *A. mellifera* honey bees, when fed a diet containing Cry9Ee toxin, no significant changes were found in the diversity and species of intestinal bacteria^48^.

The commercial products Dipel and Xentari, both containing *B. thuringiensis*, when incorporated in the honey bee Candi paste, did not affect the survival of newly emerged and foragers worker bees of *A. mellifera*. The Xentari product reduced the ability of bees to resume flight for both, newly emerged and foragers workers; however, the products did not affect other behavioral activities of *A. mellifera*, and were shown to be safe for these insects. As in the laboratory, the bees are forced to come into contact with products containing Bt, and bees fed with products based on Bt did not have significant differences in survival and evaluated behaviors, it is not necessary to take this experiment to the field, where contact conditions are less.

Although both the products showed selectivity and safety towards *A. mellifera*, it is recommended that attention should be paid to the application process, to avoid it coinciding with the workers’ foraging periods. This is important to avoid possible contamination inside the colony through transport by the foragers, and to avoid negative effects on their flight behavior. Furthermore, proper use of the products will bring benefits to the crop in terms of controlling pests of interest, maintaining the pollination carried out by *A. mellifera*, and maintaining the productivity of these honey bees.

## Material and methods

### Obtaining insects and products

Africanized honey bees (*A. mellifera*) 24-h-old (here referred to as newly emerged): frames with 19-day-old worker (pupal stage) were removed from colonies in the Honey bee breeding Laboratory (UNEPE—Apicultura) and taken to the Biological Control Laboratory where they were transferred into perforated Kraft paper bags and kept in an climate-controlled chamber (34 °C ± 2 °C, RH of 60% ± 5%) for 2–3 days until the emergence of adult worker bees.

Foragers Africanized honey bees *A. mellifera*: At the entrance of each colony, a polyvinyl chloride (PVC) cage (20 cm high × 10 cm diameter) was used to capture bees that had returned from the field. Bees from 10 colonies were used, and the bees were selected at random, so as not to interfere with the colony used. These two groups of bees were used to verify the effect of *B. thuringiensis* on survival and behavior at different ages of this insect.

The test products, Dipel and Xentari, were used at the commercial dosage recommended by the manufacturer (Table 3).

**Table 3:** Commercial products used, and the composition, dose, pests, and crops for which their use is recommended. *Source*: Agrofit^22^.

Product	Composition	Dose (P.C./ha)	Boot volume	Controlled pest	Cultures
Dipel	*Bacillus thuringiensis*, var. *kurstaki*, lineage HD-1 17.600 International Power Units per mg (minimum 27.5 billion viable spores per gram): 33.60 g/L (3.36% w/v); Inert ingredients: 966.40 g/L (96.64% m/v)	500 g/ha	200 L/ha	*Anticarsia gemmatalis*, *Pseudoplusia includens*, *Tuta absoluta*, *Heliothis virescens*, *Thyrinteina arnobia*, *Ecdytolopha aurantiana*	Cotton, citrus, eucalyptus, melon, soy, cabbage, tomato, wheat
Xentari	*Bacillus thuringiensis*, subsp. *Aizawai* equivalent to 10% (w/w) of Lepidoptera toxin: 540 g/kg (54% w/w); Inert ingredients: 460 g/kg (46% w/w)	500 g/ha	200 L/ha	*T. absoluta*, *Ascia monuste orseis*, *Plutella xylostella*, *Spodoptera frugiperda*	Tomato, broccoli cabbage, cabbage, cotton

### Survival bioassay

Newly emerged (24-h-old) worker and forager honey bees were anesthetized for until 60 s by exposure to CO_2_. The experimental unit consisted of a PVC cage (20 cm high × 10 cm diameter) containing 20 bees and enclosed with voile fabric. In addition, a diet consisting of pure Candi paste (control) or Candi paste with the incorporated treatments (Dipel and Xentari) (methodology adapted from Carvalho et al.^49^, Libardoni et al.^33^) and water-soaked cotton, which was moistened daily, was supplied. The cages were kept in a climate-controlled room (26 °C ± 2 °C, RH, 60% ± 10%, 12-h photoperiod). Each treatment consisted of 5 repetitions with 20 bees, totaling 100 bees per treatment.

The evaluation of the mortality of the bees was performed 1, 2, 3, 4, 5, 6, 9, 12, 15, 18, 21, 24, 30, 36, 42, 48, 60, 72, 96, 120, and 144 h after the incorporation of products in the feed (methodology adapted from Baptista et al.^49^, Libardoni et al.^33^. All the tests were performed in triplicates.

### Vertical displacement bioassay

After 144 h, 10 bees subjected to each treatment were chosen at random for the assessment of vertical displacement and free fall. The evaluation took place in a dark room, using a vertical tower (35 cm × 35 cm wide and 105 cm high) with a light source at the top. Inside the tower there were five levels (strata) (Table 4). For the vertical displacement test, the bees were placed at the base of the tower for 1 min, and the behavior and the maximum height reached were recorded (methodology adapted from Tomé et al.^50^).

**Table 4:** Levels (strata) for the vertical displacement and free fall (resumption of flight) test of Africanized honey bees *Apis mellifera*.

Levels (strata)	Height	
	Vertical displacement	Free fall (resumption of flight)
I	No displacement	Direct drop to the base of the tower
II	Displacement between 1 and 35 cm	Fall with resumption of flight between 1 and 35 cm
III	Displacement between 35 and 70 cm	Fall with resumption of flight between 35 and 70 cm
IV	Displacement between 70 and 105 cm	Fall with resumption of flight between 70 and 105 cm
V	Displacement direct to the light source	No fall (direct flight in the light)

Complementary to this, the free fall test was performed using the same tower. The bees were released at the top of the tower and the level at which the bee resumed flight was recorded; the levels are indicated in Table 4.

### Walking bioassay

Three PVC cages (20 cm high × 10 cm diameter) with forager bees and three PVC cages (20 cm high × 10 cm diameter) with newly emerged worker bees were prepared for each treatment (Dipel, Xentari, and control), as describe in survival bioassay. After 24 h, 14 bees from each treatment were removed and placed individually in a Petri dish (14 cm × 1.5 cm). The dishes were placed on the base of a universal support coupled to a video capture system. The behavior of each bee was recorded for 10 min and with the aid of the software Bee-move (in the registration phase) were evaluated: distance covered, walking time, resting time, and walking speed.

### Histology

After the walking behavior of the honey bees was analyzed, histological analysis of the mesenterium was performed. For this, the bees were anesthetized in a freezer (− 4 °C) for 1 min, and the mesenterium was removed and fixed in Boiun’s solution for 3 h. The samples were then washed three times in 70% alcohol and stored in a refrigerator at 4 °C until processing.

For processing, the samples were dehydrated by immersion in alcohol solutions of different concentrations using the histotechnical methodology adapted from Potrich et al.^41^. Subsequently, the samples were cleared by immersion in xylol, and the embedded in histological paraffin (histological paraffin/bee wax, 4:1). The embedded material was cut into slices (2–7 μm) by using a manual rotating microtome, and mounted on a glass slide containing albumin solution.

The sections were stained using hematoxylin and eosin. First the sections were deparaffinized, rehydrated, and washed in running water. Then, the sections were stained in hematoxylin (40 s) and eosin (10 s) and the prepared slides were covered with microscopic glass coverslips and fixed with Canada balm.

The slides containing the sections were analyzed by using a biocular biological light microscope (Zeiss Primo Star), which contains a digital camera for image capture and measurements of the villus length. Bt proteins can alter the villi and microvilli of the mesentery of insects that feed on it, so it is important to measure the intestinal villi to check for possible histological changes caused by the bacteria proteins.

### Statistical analysis

For the survival data of the workers of *A. mellifera* in the feed bioassay, a survival analysis was performed using Kaplan–Meier nonparametric estimation^51^. The K–M estimates of the treatments were compared using a pairwise log-rank test and the whole analysis was performed by using the survival package^52^ of the R software.

For the vertical displacement and free fall ordered factors data, generalized linear cumulative link models were used^53^. After the fitting the process and model checking we proceed with a post-hoc analysis using the Wald test followed by the multiple comparison Turkey test at 5% of significance. These analysis were performed using the following R packages: ordinal^54^ and emmeans^55^.

The variables related to the walk bioassay and the length of the villi are quantitative continuous numerical variables. Thus, we applied to these data a one-way ANOVA followed by the Turkey multiple comparison test at significance level of 5%. The analysis was be made through the base package of R software.
